# Case Report: Prenatal Diagnosis of a Novel Variant c.251dupT (p.N87Kfs*6) in *BCOR* Resulting in Oculofaciocardiodental Syndrome Using Whole-Exome Sequencing

**DOI:** 10.3389/fgene.2022.829613

**Published:** 2022-03-25

**Authors:** Jianlong Zhuang, Chunnuan Chen, Yu’e Chen, Shuhong Zeng, Yuying Jiang, Yuanbai Wang, Xinying Chen, Yingjun Xie, Gaoxiong Wang

**Affiliations:** ^1^ Prenatal Diagnosis Center, Quanzhou Women’s and Children’s Hospital, Quanzhou, China; ^2^ Department of Neurology, The Second Affiliated Hospital of Fujian Medical University, Quanzhou, China; ^3^ Ultrasonography, Quanzhou Women’s and Children’s Hospital, Quanzhou, China; ^4^ Department of Obstetrics and Gynecology, Key Laboratory for Major Obstetric Diseases of Guangdong Province, The Third Affiliated Hospital of Guangzhou Medical University, Guangzhou, China; ^5^ Key Laboratory of Reproduction and Genetics of Guangdong Higher Education Institutes, The Third Affiliated Hospital of Guangzhou Medical University, Guangzhou, China; ^6^ Quanzhou Women’s and Children’s Hospital, Quanzhou, China

**Keywords:** oculofaciocardiodental, chromosomal microarray analysis, whole-exome sequencing, BCOR, frameshift mutation, hemizygous variant

## Abstract

**Background:** Oculofaciocardiodental (OFCD) syndrome is an X-linked dominant syndrome caused by BCOR *variants*, which manifests only in females and presumed leading to male lethality. Herein, we aim to present a prenatal diagnosis for OFCD syndrome associated with a novel hemizygous variant in *BCOR* gene.

**Case presentation:** A 29-year-old pregnant woman from Quanzhou Fujian Province, China, with fetal ultrasound anomalies, was enrolled in this study. A normal 46, XY karyotype with no abnormalities was observed in the fetus detected on microarray. Furthermore, a whole-exome sequencing (WES) detection result demonstrated that a novel hemizygous variant of c.251dupT (p.N87Kfs*6) in the *BCOR* gene was identified in the fetus, which was a frameshift mutation and classified as a likely pathogenic variant, and may lead to OFCD syndrome according to the clinical feature of the fetus. In this case, male lethality had not occurred by the end of the second trimester, then termination of the pregnancy was conducted at a gestational age of 26 weeks. Sanger sequencing of parental samples revealed that the variant was maternally transmitted, which was consistent with the OFCD syndrome phenotypic features observed in her.

**Conclusions:** In the study, we first present the affected male with a novel variant in *BCOR* that leads to the OFCD syndrome. Additionally, our study broadened the spectrum of *BCOR* results in the OFCD syndrome and provided the valuable references for prenatal genetic consultation.

## Introduction

With the continuous application and development of high-throughput sequencing technology, whole-exome sequencing (WES) based on next-generation sequencing technology has been increasingly used in scientific research and clinical diagnosis. The human exome contains about 180,000 exons, accounting for only 1% of the whole human genome; however, around 85% of the variants related to diseases exist in the exon region (Choi et al., 2009; Ng et al., 2010). Recent studies have shown that variants in a single gene would exhibit fetal ultrasound abnormalities *in utero*, with normal karyotype and chromosomal microarray analysis results. An additional pathogenic mutation detection rate of 6.2%–80.0% was observed by prenatal WES detection over chromosomal microarray analysis (CMA) detection (Best et al., 2018; Lord et al., 2019; Petrovski et al., 2019). Therefore, it is of great value using WES technology to investigate pathogenic mutations of fetal ultrasonic structural abnormalities at a single-gene level.

Pathogenic variants in the BCL-6 corepressor (*BCOR*, OMIM: 300485) on chromosome Xp11.4 will result in two distinct syndromes including oculofaciocardiodental syndrome (OFCD, OMIM: 300166) and Lenz microphthalmia syndrome (OMIM: 309800) (Ng et al., 2004). OFCD syndrome is a rare X-linked dominant genetic disorder, which typically affects females and is presumed to lead to male lethality caused by a variety of *BCOR* null mutations including deletional, nonsense, splicing, truncating, and frameshift mutations (Wilkie et al., 1993; Ragge et al., 2019). It is characterized by congenital cataract, dental anomalies, skeletal abnormalities, cardiac septal defect, cleft palate, etc. (Ng et al., 2004; Hilton et al., 2009). In contrast, Lenz microphthalmia syndrome is an X-linked recessive inheritance pattern, which showed normal clinical phenotype in females, and only affected males with microphthalmia, intellectual disability, skeletal and urogenital malformations, and other anomalies. While a previous study conducted by Horn et al. (Horn et al., 2005) indicated that the *BOCR* gene may not be the major gene in the Lenz microphthalmia syndrome, to date, only one specific missense mutation of c.254C > T (p.P85L) in *BCOR* has been reported to associate with the Lenz microphthalmia syndrome (Temtamy et al., 2000; Ersin et al., 2003).

To date, only a previous report, which referred to a prenatal diagnosis analysis of the Lenz microphthalmia syndrome associated with the typical mutation of c.254C > T, was conducted in 2013 (Suzumori et al., 2013). No report of prenatal diagnosis analysis of X-linked dominant OFCD syndrome relevant to the *BCOR* gene was observed. In this study, we report the first case of prenatal diagnosis for the OFCD syndrome in an affected male with a novel frameshift mutation in the *BCOR* gene.

## CASE PRESENTATION

A 29-year-old gravida 2, para 1 pregnant woman from Quanzhou Fujian Province, China, referred to the Prenatal Diagnosis Center of Quanzhou Women’s and Children’s Hospital at the gestational age of 16 + 2 weeks. Her husband was 31 years old, and the couple denied any family history of inheritance disease and consanguinity. At her first pregnancy, a female infant was delivered at the gestational age of 39 + 6 weeks in 2019. At present, she is 2.5 years old with a normal phenotype. At this pregnancy, the second trimester Down’s screening was performed, and moderate risk of trisomy 21 (1/552) was observed. The subsequent noninvasive prenatal testing test results elicited a low risk of T21, T18, and T13. However, ultrasonic examination conducted at 17 + 6 weeks of gestation suggested the possibility of fetal duodenal obstruction and a variety of soft index abnormalities, including an enhanced echo of fetal renal parenchyma and punctate hyperechoic of the left ventricle.

**FIGURE1:**
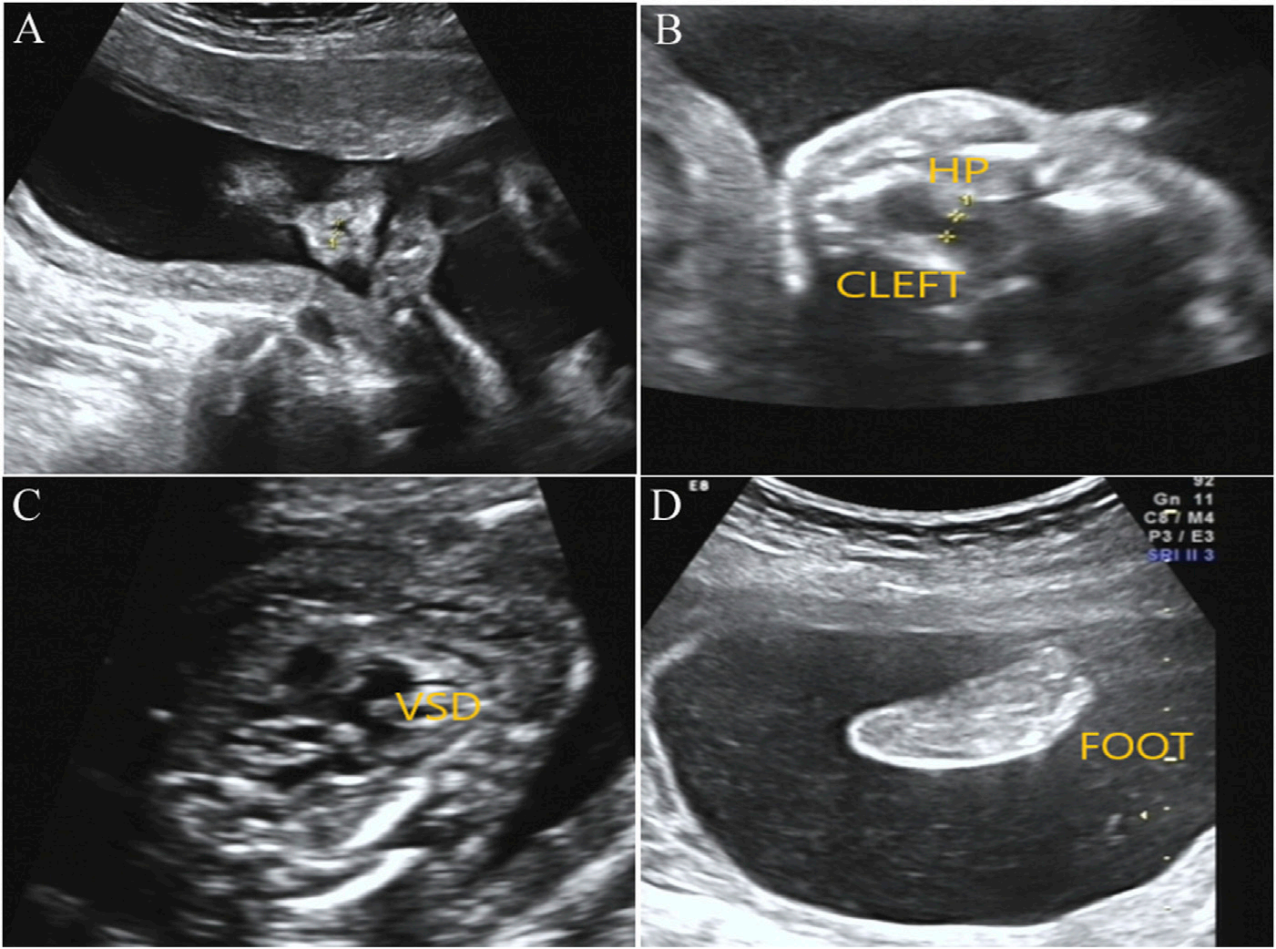
Prenatal ultrasound examination results in the fetus with *BOCR* variant. **(A)** Ultrasound detection results showed right alar fissure in the fetus. **(B)** Fetal ultrasound results indicated continuous interruption of cleft palate. HP, hard palate. **(C)** Fetal ultrasound results elicited fetal ventricular septal defect (VSD). **(D)** Toe syndactyly was also observed in the fetus by ultrasound examination.

After genetic counseling and informed consent, amniocentesis was performed at 20 weeks. Karyotype analysis combined with CMA was used to detect fetal chromosomal abnormalities and copy number variants, while no obvious abnormalities were found. At the gestational age of 24 weeks, a three-dimensional color Doppler ultrasound was performed and indicated several fetal structure anomalies including fetal right nasal fissure, duodenal obstruction, cleft palate, ventricular septal defect, and toe syndactyly (Figure 1).

**FIGURE2:**
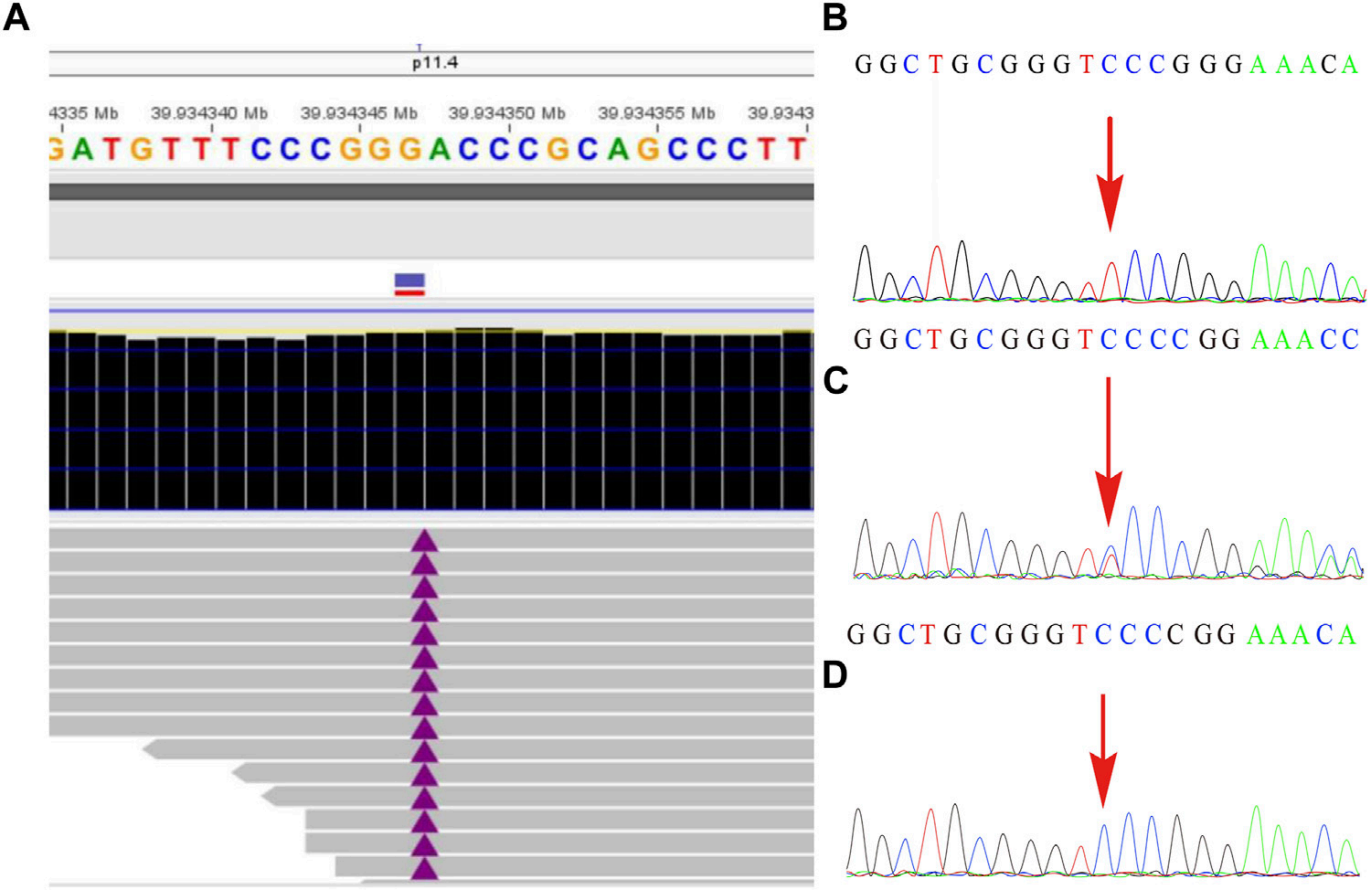
The variant in the *BCOR* gene was detected by whole-exome sequencing and further verified by Sanger sequencing. **(A)** A frameshift mutation c.251dupT (p.N87Kfs*6) of the *BCOR* gene in the fetus was detected by WES technology. **(B)** shows the hemizygosity of the variant in the affected fetus, while **(C)** demonstrated that the mother was heterozygous and **(D)** showed that the frameshift variant was not present in his father.

The remaining amniotic fluid was used for DNA extraction and further WES detection. The WES detection result delineated a novel hemizygous variant of c.251dupT (p.N87Kfs*6) in exon 4 of the *BCOR* gene, which was identified in the male fetus (Figure 2). It was a frameshift mutation and classified as a likely pathogenic variant according to the ACMG guidelines (Richards et al., 2015), with no frequency that has been reported in databases including gnomAD, 1000 genomes, dbSNP, Clinvar, ExAC, as well as PubMed databases. According to the variant type and fetal clinical phenotypes, the frameshift mutation in the *BCOR* gene may lead to OFCD syndrome. Male lethality was not observed by the end of the second trimester, then termination of pregnancy was conducted at the gestational age of 26 weeks. Segregation analysis indicated that the variant in the *BCOR* gene was inherited from his mother who exhibited a phenotype associated with OFCD syndrome including long, thin face, flat nasal bridge, broad nasal tip, high palate, microphthalmia, dental anomalies (teeth are crowded and irregularly arranged), and ventricular septal defect, but with normal mental and physical development and without congenital cataract. Moreover, the novel variant in the *BCOR* gene was absent in the proband’s sister who exhibits a normal clinical phenotype.

## Discussion and conclusion

Variants of the *BCOR* gene will result in two distinct syndromes including the OFCD syndrome and Lenz microphthalmia syndrome. A prenatal diagnosis analysis for the Lenz microphthalmia syndrome associated with typically missense mutation of c.254C > T was also identified (Suzumori et al., 2013). To date, no information is available on prenatal diagnosis analysis of the OFCD syndrome that associates with the *BCOR* gene variants. Here, the first case of prenatal diagnosis for the OFCD syndrome with a novel frameshift mutation c.251dupT (p.N87Kfs*6) in exon 4 of the *BCOR* gene was identified. Moreover, this was also the first case of a male fetus who carried the *BCOR* mutation that resulted in OFCD syndrome to the best of our knowledge. It is worth noting that for the affected male fetus, the mother was still undergoing pregnancy at 26 weeks of gestation.

OFCD syndrome is typically caused by *BCOR* variants that lead to premature termination codons, including frameshift mutations in the form of small deletions or duplications, or microdeletions in the *BCOR* gene. The Lenz microphthalmia syndrome is usually caused by missense mutations, which only lead to changes in amino acids. In the present study, we report a novel frameshift variant of c.251dupT (p.N87Kfs*6) in exon 4 of the *BCOR* gene in a male fetus, and ultrasound examination results showed that the fetus had several fetal structure anomalies including fetal right nasal fissure, duodenal obstruction, cleft palate, ventricular septal defect, and toe syndactyly. This hemizygous variant has never been reported and has no frequency in the database, which was classified as a likely pathogenic variant according to the ACMG guidelines (PVS1 + PM2). Additionally, the fetus’ mother harbored the same variant and exhibited a phenotype associated with the OFCD syndrome including facial deformity, microphthalmia, dental anomalies, and ventricular septal defect. According to the inheritance pattern and the clinical phenotypes in the fetus and his mother, we believe that the novel frameshift mutation in *BCOR* would lead to the OFCD syndrome.

Phenotypic variability was also present in the OFCD syndrome and shows different clinical symptoms in the same family (Lozić et al., 2012). A previous study conducted by Davoody et al. elicited a heterozygous frameshift variant of c. 2858_2859delAA (p.K593SfsX7) in exon 4 of the *BCOR* gene was identified in a female patient with characteristic facial features, while no indication of atrial septal defect or ventricular septal defect existed (Davoody et al., 2012). Additionally, a novel mutation c.265G > A on exon 4 was identified in a Japanese female and diagnosed as OFCD syndrome that exhibits clinical phenotypes including congenital cataract, ventricular septal defect, dental deformity, and without cleft palate (Kato et al., 2018). The largest study (Hilton et al., 2009) reported 34 female patients in 20 families with variants of the *BCOR* gene exhibiting the OFCD syndrome. All of the patients had congenital cataract, and microphthalmia and/or microcornea that were observed in 28 cases. In contrast, the study conducted by Michelle et al. (Hamline et al., 2020) showed that 55% (23/42) of OFCD animals had lens opacification (indicative of cataracts), and 35% (8/23) were affected bilaterally, which showed clinical diversity of ocular deformity. In the present case, the mother did not have a cataract feature and cleft palate, while a high palate was observed. Moreover, *BCOR* hemizygosity mouse model showed early male embryo lethality by E9.5 (Cox et al., 2010; Hamline et al., 2020). Interestingly, in our study, the mother of the affected male fetus was still undergoing pregnancy at 26 weeks of gestation. Moreover, more work needs to be done to determine whether the male embryo with the presented variant in the *BCOR* gene will lead to lethality in the third trimester.

In conclusion, a prenatal diagnosis was first conducted eliciting a novel frameshift mutation c.251dupT (p.N87Kfs*6) in exon 4 of the *BCOR* gene and resulted in the OFCD syndrome. Moreover, the affected male fetus of the OFCD syndrome was first reported, and the pregnancy was still ongoing at the end of the second trimester. Our study provides valuable data for prenatal genetic consultation of OFCD syndrome and further strengthened the application value of WES in prenatal diagnosis.

## Data Availability

The raw data supporting the conclusion of this article will be made available by the authors, without undue reservation.
